# Assessing the Impact of Patient-Facing Mobile Health Technology on Patient Outcomes: Retrospective Observational Cohort Study

**DOI:** 10.2196/19333

**Published:** 2020-06-26

**Authors:** Courtenay R Bruce, Patricia Harrison, Tariq Nisar, Charlie Giammattei, Neema M Tan, Caitlin Bliven, Jamie Shallcross, Aroub Khleif, Nhan Tran, Sayali Kelkar, Noreen Tobias, Ana E Chavez, Dana Rivera, Angela Leong, Angela Romano, S Nicholas Desai, Josh R Sol, Kayla Gutierrez, Christopher Rappel, Eric Haas, Feibi Zheng, Kwan J Park, Stephen Jones, Paul Barach, Roberta Schwartz

**Affiliations:** 1 System Quality & Patient Safety Houston Methodist Hospital System Houston, TX United States; 2 Center for Outcomes Research Houston Methodist Research Institute Houston, TX United States; 3 CareSense Philadelphia, PA United States; 4 Information Technology Department Houston Methodist Hospital Houston, TX United States; 5 Information Technology Portfolio Management Office Houston, TX United States; 6 Cardiovascular Surgery Associates Houston Methodist Hospital Houston, TX United States; 7 Houston Methodist Orthopedic and Sports Medicine - The Woodlands Houston Methodist The Woodlands The Woodlands, TX United States; 8 Clinical Design and Planning Houston Methodist Hospital System Houston, TX United States; 9 Houston Methodist Specialty Physician Group Houston Methodist Orthopedics & Sports Medicine Houston Methodist Hospital Houston, TX United States; 10 Department of Orthopedics Houston Methodist Hospital Houston, TX United States; 11 Center for Innovation Houston Methodist Hospital Houston, TX United States; 12 Houston Methodist Sugar Land Hospital Houston, TX United States; 13 Department of Surgery Houston Methodist Hospital Houston, TX United States; 14 Jefferson College of Population Health Thomas Jefferson University Philadelphia, PA United States

**Keywords:** mHealth, patient-centered care, patient satisfaction, length of stay, patient activation, patient empowerment, patient engagement, patient involvement, hospital stay, communication programs

## Abstract

**Background:**

Despite the growth of and media hype about mobile health (mHealth), there is a paucity of literature supporting the effectiveness of widespread implementation of mHealth technologies.

**Objective:**

This study aimed to assess whether an innovative mHealth technology system with several overlapping purposes can impact (1) clinical outcomes (ie, readmission rates, revisit rates, and length of stay) and (2) patient-centered care outcomes (ie, patient engagement, patient experience, and patient satisfaction).

**Methods:**

We compared all patients (2059 patients) of participating orthopedic surgeons using mHealth technology with all patients of nonparticipating orthopedic surgeons (2554 patients). The analyses included Wilcoxon rank-sum tests, Kruskal-Wallis tests for continuous variables, and chi-square tests for categorical variables. Logistic regression models were performed on categorical outcomes and a gamma-distributed model for continuous variables. All models were adjusted for patient demographics and comorbidities.

**Results:**

The inpatient readmission rates for the nonparticipating group when compared with the participating group were higher and demonstrated higher odds ratios (ORs) for 30-day inpatient readmissions (nonparticipating group 106/2636, 4.02% and participating group 54/2048, 2.64%; OR 1.48, 95% CI 1.03 to 2.13; *P*=.04), 60-day inpatient readmissions (nonparticipating group 194/2636, 7.36% and participating group 85/2048, 4.15%; OR 1.79, 95% CI 1.32 to 2.39; *P*<.001), and 90-day inpatient readmissions (nonparticipating group 261/2636, 9.90% and participating group 115/2048, 5.62%; OR 1.81, 95% CI 1.40 to 2.34; *P*<.001). The length of stay for the nonparticipating cohort was longer at 1.90 days, whereas the length of stay for the participating cohort was 1.50 days (mean 1.87, SD 2 vs mean 1.50, SD 1.37; *P*<.001). Patients treated by participating surgeons received and read text messages using mHealth 83% of the time and read emails 84% of the time. Patients responded to 60% of the text messages and 53% of the email surveys. Patients were least responsive to digital monitoring questions when the hospital asked them to *do* something, and they were most engaged with emails that *did not* require action, including informational content. A total of 96% (558/580) of patients indicated high satisfaction with using mHealth technology to support their care. Only 0.40% (75/2059) patients *opted-out* of the mHealth technology program after enrollment.

**Conclusions:**

A novel, multicomponent, pathway-driven, patient-facing mHealth technology can positively impact patient outcomes and patient-reported experiences. These technologies can empower patients to play a more active and meaningful role in improving their outcomes. There is a deep need, however, for a better understanding of the interactions between patients, technology, and health care providers. Future research is needed to (1) help identify, address, and improve technology usability and effectiveness; (2) understand patient and provider attributes that support adoption, uptake, and sustainability; and (3) understand the factors that contribute to barriers of technology adoption and how best to overcome them.

## Introduction

### Background

Improving patient safety and quality of care remains to be the key goal of health care systems. In 2001, the Institute of Medicine identified patient-centered care as a health care quality indicator [1,2]. Within the past decade, top health care organizations have embraced mobile health (mHealth) as part of their patient-centered initiatives and their drive to achieve the quadruple aim [3-7]. Indeed, 18 of the 20 hospitals listed on the 2019-2020 Honor Roll for the US News and World Report have adopted at least one patient-centered mHealth technology [3]. Funding for mHealth technologies exceeded US $3 billion in the United States in the first 6 months of 2019 [4], and the European Union has committed to investing US $24 billion in mHealth technologies [5,6]. In spring 2020, coronavirus disease 2019 (COVID-19) resulted in the rapid global uptake of patient-facing mHealth technologies to support patient needs and protect health care providers while promoting social distancing [7].

Despite this growth, there remains a paucity of data regarding whether mHealth technologies have scientific merit and are effective to warrant such widespread implementation [8]. Specifically, it is unclear whether mHealth technologies positively impact *clinical outcomes*, such as hospital length of stay, patient readmissions, or complications [9]. It is also unclear whether mHealth technologies impact *patient-centered care outcomes*, defined and measured by the perceptions of patient experiences, patient engagement, or patient satisfaction [10,11]. Existing studies focus on telemonitoring mHealth technologies and clinical outcomes, often showing unremarkable results. For instance, one large-scale study showed no reduction in readmission rates [12], and a few single-institution assessments demonstrated that telemonitoring using mHealth technologies could modestly improve patient adherence [13,14]. mHealth technologies beyond telemonitoring, such as tele-education, teleconsultation, and digital navigation, are largely unexplored and have not shown meaningful impacts on patient outcomes [5-14].

### Objectives

We sought to assess whether an innovative mHealth technology system with several overlapping purposes, specifically tele-education and telemonitoring features, can impact (1) clinical outcomes (ie, readmission rates, revisit rates, and length of stay) and (2) patient-centered care outcomes (ie, patient engagement, patient experience, and patient satisfaction).

## Methods

### Setting and Context

This study was a retrospective, observational cohort study. We retrospectively analyzed all patients treated by orthopedic surgeons in our hospital system who actively participated in using mHealth technology from January 1 to December 31, 2019, and compared them with all patients of nonparticipating orthopedic surgeons during the same period. The implementation phase was staggered in a phased rollout. Surgeons who were not yet approached did not participate, and the technology was not offered to their patients. All patients in both groups underwent a primary total joint (hip or knee) replacement (TJR). This observational cohort study was approved by the hospital system’s institutional review board. The hospital system consists of one 2264-bed tertiary academic medical center located in Houston, Texas, along with 7 community hospitals (300-700 beds) in the suburbs of Houston, Texas.

We excluded patients from the analysis who indicated any language other than English as their preferred language (percentages of which are reported in the Results section) to mitigate selection bias. We compared patients who only preferred the English language and used mHealth technology with all patients who preferred the English language and used mHealth technology.

We also conducted an uptake analysis of the first few months in which mHealth technology was available (January 1, 2019-April 30, 2019). Our goal in conducting a subset analysis was to show a robust biological gradient [15]. The presence of a dose-response relationship supports the causal association between an exposure and an effect and durability of the results, and, by extension, we sought to show the external generalizability benefits to the overall population [15,16].

### The Participating Cohort

The technology consisted of a digital education and monitoring platform, CareSense, (MedTrak, Inc) for patients using their computers or mobile devices with text and email messages in English about their medical condition. Text messages were automatically delivered over the cellular network (SMS texts) via an internet connection. Patients did not need to download an mHealth app or go to a web-based patient portal for emails and texts to be transmitted. Patients did have the option, however, to access a secure, web-based portal to receive all messages in one area.

The text messages were converted to automated phone calls in cases where patients did not have text messaging capabilities. One or two messages were sent each day for the 20 days before surgery, were stopped when the patient was admitted to the hospital, and resumed once or twice a day for 30 days following the patient’s hospital discharge. We refer in this paper to the full sequence of messages throughout the 50-day period as *the pathway*.

### Mobile Health Technology Content

The content of *the pathway* was primarily designed to achieve several purposes: (1) provide education, (2) monitor health and recovery, (3) provide key reminders to needed actions or taking of medication, and (4) ensure resolution of patients’ action items (Table 1).

**Table 1:** Example of patient messages and clinical domains.

Period the message was sent and purpose of the message		Example messages	Clinical domains or goals	Response rate (%)
**Presurgery**				
	Educating	Questions to ask your physician about preparing your home for surgery	Promoting preparation and understanding	94
	Closing action items	“Have you scheduled your visit for presurgery lab work?” and “Have you completed your presurgical clearance paperwork?”	Promoting adherence and self-management	58
	Monitoring	“Have you checked your hemoglobin A_1C_? Was it over a 7?”	Optimizing health in preparation for surgery	73
	Closing action items	“Do you have someone who can pick you up after surgery, regardless of what hour you are discharged?”	Discharge planning	87
	Closing action items	“Do you have someone available after surgery who can help you for the first 24-48 hours?”	Enlisting help of social supports	87
**Postsurgery**				
	Educating	Tips on how to take care of your wound when to call our office ASAP^a^	Monitoring and managing symptoms	88
	Educating	Tips on mobility exercises	Optimizing health following surgery	89
	Closing action items	“Have you scheduled your follow-up appointment yet?”	Outpatient follow-up	83
	Monitoring	“Take a look at your incision site. Is it sore, very red, puffy…?”	Monitoring and managing symptoms	89

^a^ASAP: as soon as possible.

Generally, time-sensitive and short messages were sent via text messaging, typically consisting of alphabetic and numeric characters, and longer educational messages were sent via email. Table 1 demonstrates examples of messages for all 4 purposes.

The messages were unidirectional or bidirectional. For unidirectional messages, there was no expectation of patient response. Unidirectional messages were educational or informative in nature. The bidirectional messages were sent to solicit patient responses using close-ended questions (Table 1). The bidirectional messages allowed clinicians to monitor patients’ health and recovery or, alternatively, to ensure that the patient completed important action items before and/or following their surgery.

When patients responded to bidirectional messages in a concerning way, an alert was automatically generated and routed to their health care professionals. For example, one bidirectional message read, “Please identify your pain level. Press 1 for no pain or mild pain (1-3 on a pain scale); Press 2 for moderate pain (4-6 on a pain scale); Press 3 for severe pain (7-8 on a pain scale); Press 4 for extreme pain (9-10 on a pain scale).” A patient who responded by pressing the numbers 3 or 4 generated an alert that was sent to the health care team (ie, medical assistants [MAs] or nurses) via email, letting them know that a patient had responded to a monitoring question that required their timely response.

### Procedures

During the surgery scheduling process, hospital schedulers of participating surgeons asked all English-speaking patients undergoing TJR whether they would be willing to receive digital messages. If so, the scheduler used the electronic medical record to activate *the pathway*. Clinicians and schedulers did not need to push messages to patients, as all messages were transmitted automatically via text/phone calls and email once the scheduler activated *the pathway*.

Patients could *opt-out* at any time after enrollment by typing the word *stop* in response to any text messages to stop receiving email and text messages. For patients who did not have text messaging abilities, they could press a number on their telephone number pad in response to phone calls to stop all phone calls and email messages.

The education provided to patients was minimal, largely because they did not need to download an app or go to a portal to receive messages. The messages were transmitted automatically for patients who agreed to receive them, requiring little technical expertise on the part of the patients. Patients were directed to call one hospital-based employee who had content and technical expertise with any questions about mHealth technology.

### Outcome Measures

#### Readmissions and Revisit Rates

Hospital readmissions were defined as any subsequent unplanned inpatient admission to any of our system-based acute care facilities occurring within 30, 60, and 90 days of hospital discharge following the qualifying total joint operations. Only unplanned inpatient admissions (for any cause) to short-term acute care, excluding transfer encounters, qualified as readmission for the study, as our inclusion and exclusion criteria for calculation were consistent with the Centers for Medicaid and Medicare Services specifications and methodological standards [17].

The patient revisit rates were defined as any visit to an acute care facility that occurred within 30, 60, and 90 days after discharge following a qualifying total joint operation, aside from unplanned inpatient admissions—namely, emergency department visits; unplanned, unscheduled outpatient visits; and observation status visits [17].

The hospital readmissions and revisit rates were calculated based on reviewing electronic medical records for all elective primary total knee or total hip replacement operations performed between January 1 and December 31, 2019. Patients who were readmitted to the same hospital on the same calendar day of discharge for the same diagnosis as the index admission were considered to have 1 single continuous admission (ie, 1 index admission), and patients who were readmitted for a different condition from the index admission were considered to have a readmission within the measure. The analysis excluded staged surgical procedures by identifying the patients who were readmitted for another primary hip or knee procedure within the 30-, 60-, or 90-day periods.

#### Length of Stay

The hospital length of stay comprised the entire length of hospitalization and was calculated using the admission date until the discharge date.

#### Other Prespecified End Points

Patient-centered care outcomes included patient engagement, patient experience, and patient satisfaction. *Patient engagement* was defined as the degree to which the patient engaged with the mHealth technology [18,19]: (1) minimal engagement (ie, the patient read ≤25% of all messages and responded to ≤25% of all messages in *the pathway*, as indicated by a read receipt), (2) moderate engagement (ie, the patient read 26%-50% of all messages and responded to 26%-50% of all messages), or (3) high engagement (ie, the patient read ≥51% of all messages and responded to ≥51% of all messages). The patient had to read ≥51% of all messages and respond to ≥51% of all messages to be considered highly engaged. If they read messages but did not respond at a level of ≥51%, they would not meet the engagement threshold. We chose these thresholds consistent with the literature on patient engagement, where researchers proposed that empirical thresholds of engagement with mHealth technology must be met to show sufficient engagement [8,19].

*Patient experience* was defined as any process observable by patients, including their subjective experiences (eg, quality of communication) and their objective experiences (eg, how often communication occurred) [20]. We analyzed patient experiences by evaluating the patients’ responses to the validated, reliable Hospital Consumer Assessment of Healthcare Providers and Systems (HCAHPS) survey [21], regarding 4 questions:

1. Before giving you any new medicine, how often did the hospital staff describe possible side effects in a way you could understand? (4-point Likert scale, never to always)
2. How often did the hospital staff tell you what the medicine was for? (4-point Likert scale, never to always)
3. Did doctors, nurses, or other hospital staff talk with you about whether you would have the help you needed when you left the hospital? (yes or no)
4. Did you get information in writing about what symptoms or health problems to look out for after you left the hospital? (yes or no)

We compared the experience scores for each of these 4 questions for patients in the participating group as compared with those in the nonparticipating group.

*Patient satisfaction* was defined as whether patients’ expectations were met [10]. For the participating cohort, we analyzed the patients’ collective responses (Likert scale 1-5: strongly agree to strongly disagree) to a text question that we embedded in *the pathway* which asked, “How much do you agree with the following statement: It was helpful for me to receive reminders and emails from this program.” We chose this question because questions on helpfulness and ease of use are considered the most frequently used questions in most validated instruments for patient satisfaction [9,19,20].

### Data Analysis

We used the two-tailed *t* test and Wilcoxon test for continuous variables and chi-square test for categorical variables to compare the baseline characteristics between the 2 groups [16]. The data about patient demographics were available for 100% of patients and providers. The clinical end points were compared using chi-square tests. Logistic regression models using the generalized estimated equation (GEE) method accounting for repeated measurements were performed on the categorical outcomes and the gamma-distributed model of the continuous variable.^ ^All tests for significance were two-tailed, using an alpha level of .05, and 95% CIs were provided. All statistical analyses were performed using R version 3.6.0 [21]. A multivariate regression analysis was conducted to minimize confounding factors and their potential impacts, such as age, gender, and comorbidities.

## Results

### Diversity of the Sample

#### Patients

A total of 2059 patients who underwent TJR were treated by participating surgeons using mHealth technology from January to December 2019, whereas 2554 patients who underwent TJR were treated by nonparticipating surgeons who did not use mHealth technology during the same period. To minimize the possibility of language being a confounding variable in the study, 162 patients were excluded from the analysis, including 119 patients who were not enrolled with mHealth technology who indicated a preference for a language other than English, and 43 patients who were enrolled in the mHealth technology who indicated a preference for a language other than English. This resulted in 2059 patients who underwent TJR treated by participating surgeons and 2554 patients who underwent TJR treated by nonparticipating surgeons. Only 0.30% (59/19,667) patients declined to enroll in the mHealth technology program.

Separately, we also conducted an analysis of the first few months when patients who underwent TJR treated by participating surgeons were offered mHealth technology (January 1, 2019-April 30, 2019) as compared with patients of nonparticipating surgeons. These results, which were similar to the January 1 to December 31, 2019, outcomes on all measures, can be found in Multimedia Appendix 1.

#### Demographics

The differences in the baseline characteristics of the patients are described in Table 2. Overall, the mean age of the patients was 68 years for the nonparticipating group and 67 years for the participating groups (*P<*.05). There were 57.75% (1475/2554) of females in the nonparticipating group and 60.42% (1244/2059) in the participating group (*P*=.07). The preferred language, ethnicity, and race were nearly identical in the nonparticipating and participating cohorts (Table 2).

**Table 2:** Sample patient characteristics of nonparticipating and participating groups.

Variables		Nonparticipating cohort (n=2554)	Participating cohort (n=2059)	*P* value
Age (years), mean (SD)		68.07 (9.76)	66.83 (9.63)	*<.05* ^a^
**Sex, n (%)**				.07
	Female	1475 (57.75)	1244 (60.42)	
	Male	1079 (42.25)	815 (39.58)	
**Race, n (%)**				
	White	2100 (82.22)	1687 (81.93)	.83
	African American	332 (13.00)	263 (12.77)	.85
	Asian	54 (2.11)	49 (2.38)	.61
	Native American	6 (0.23)	0 (0.00)	.50
	Others	28 (1.10)	14 (0.68)	.19
**Ethnicity, n (%)**				
	Hispanics	175 (6.85)	133 (6.46)	.63
	Non-Hispanics	2360 (92.40)	1913 (92.91)	.55
	Declined to answer	19 (0.74)	1297 (0.63)	.78
**Comorbidities, n (%)**				
	Hypertension	829 (32.46)	792 (38.47)	*<.05*
	Hyperlipidemia	578 (22.63)	566 (27.49)	*<.0*
	Unilateral primary osteoarthritis	1112 (43.54)	1205 (58.52)	*<.001*
	Gastroesophageal reflux disease	445 (17.42)	4410 (21.42)	*<.05*

^a^The italicized values are statistically significant at a level of .05.

The surgeon demographics were similar between the nonparticipating and participating cohorts, and the differences were almost negligible, except for 1 fundamental attribute: the nonparticipating surgeons had higher baseline (ie, pre-mHealth technology) patient satisfaction scores compared with the participating surgeons (22/22, 100% compared with 92% baseline patient satisfaction scores, respectively; *P*<.05; Table 3).

**Table 3:** Comparison between participating surgeons and nonparticipating surgeons.

Variables	Participating surgeons (n=22)	Nonparticipating surgeons (n=25)	*P* value
Experience (years), mean (SD)	19.73 (12.53)	22.56 (9.23)	.39
Case volume, median (IQR)	166.5 (66.75-258.5)	107 (18.5-181.25)	.13
Readmission, median (IQR)	2.02 (1.65-2.31)	2.10 (1.10-3.1)	.97
Patient satisfaction scores, median (IQR)	92 (88.25-96)	100 (92-100)	*<.05* ^a^

^a^The italicized values are statistically significant at a level of .05.

#### Patient Comorbidities

The comorbidities differed in both groups (Table 2), with the participating group having more comorbidities at baseline (ie, before enrolling them in *the pathway*). Specifically, 32.46% (829/2554) of the patients in the nonparticipating group were treated for hypertension, with 38.47% (792/2059) of patients in the participating group treated for the same condition (*P*<.05). The incidence of hyperlipidemia occurred at a higher rate among patients in the participating group than in the nonparticipating group (566/2059, 27.49% vs 578/2554, 22.63%, respectively; *P*<.05). Other comorbidities included gastroesophageal reflux diseases, unilateral primary osteoarthritis of the right hip, and unilateral primary osteoarthritis left knee, which also impacted patients within the participating group at higher rates than those in the nonparticipating group (4410/2059, 21.42% participating vs 445/2554, 17.42% nonparticipating for reflux; **P*<.05* and 1205/2059, 58.52% participating vs 1112/2554, 43.54% nonparticipating for osteoarthritis; *P*=.03).

### Outcome Measures

#### Readmissions and Revisit Rates

Within 30 days of surgery, 106/2636, 4.02% of inpatient readmissions occurred for the nonparticipating group, and 54/2048, 2.64% of inpatient readmissions occurred for the participating group (*P*=.001; Table 4).

**Figure 1:**
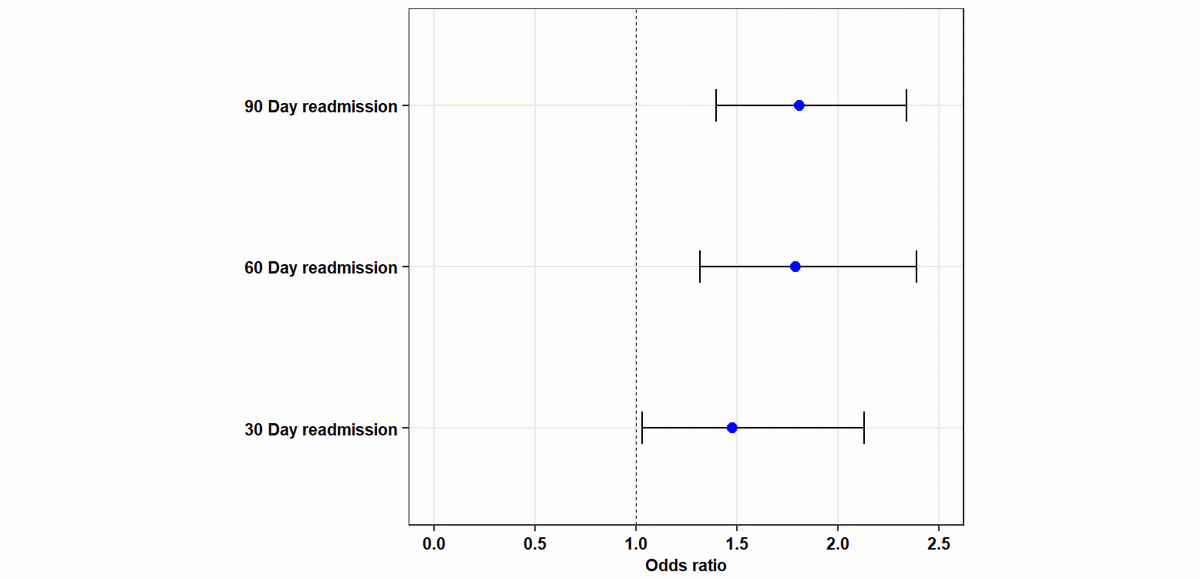
Odds ratio for participating versus nonparticipating patients. The bars represent 95% CIs and the dots represent the odds ratio.

Within 60 days after surgery, 194/2636, 7.36% inpatient readmissions occurred in the nonparticipating group, and 85/2048, 4.15% inpatient readmissions occurred in the participating group (*P*<.001). Within 90 days after surgery, 261/2636, 10.00% of inpatient readmissions occurred in the nonparticipating group, and 115/2048, 5.62% of inpatient readmissions occurred in the participating group (*P*<.05). After adjusting for demographics and comorbidities (hypertension, hyperlipidemia, gastroesophageal disease, and osteoarthritis), a multivariate logistic model (GEE) demonstrated that the nonparticipating group had a higher odds ratio (OR) for 30-day inpatient readmissions (OR 1.48, 95% CI 1.03 to 2.13; *P*=.04), 60-day inpatient readmissions (OR 1.79, 95% CI 1.32 to 2.39; *P*<.001), and 90-day inpatient readmission rates (OR 1.81, 95% CI 1.40 to 2.34; *P*<.001) when compared with the participating group (Figure 1). The patient revisit rates are shown in Table 4.

**Table 4:** Hospital readmissions and revisit rates analyses.

Outcome variable^a,b^		Control		Intervention		*P* value
		N	Value, n (%)	N	Value, n (%)	
**Hospital readmission**						
	30 days	2636	106 (4.02)	2048	54 (2.64)	*.01* ^c^
	60 days	2636	194 (7.36)	2048	85 (4.15)	*<.001*
	90 days	2636	261 (9.90)	2048	115 (5.62)	*<.05*
**Emergency department visits**						
	30 days	2594	134 (5.17)	2081	85 (4.08)	.10
	60 days	2594	183 (7.05)	2081	120 (5.76)	.09
	90 days	2594	206 (7.94)	2081	146 (7.02)	.26
**Unplanned, unscheduled outpatient visits**						
	30 days	2571	24 (0.93)	2064	18 (0.87)	.95
	60 days	2571	81 (3.15)	2064	52 (2.52)	.23
	90 days	2571	129 (5.02)	2064	77 (3.73)	*.04*
**Observation status visits**						
	30 days	2556	60 (2.35)	2059	26 (1.26)	*.01*
	60 days	2556	95 (3.72)	2059	36 (1.75)	*<.05*
	90 days	2556	125 (4.89)	2059	49 (2.38)	*<.05*

^a^Hospital readmissions were defined as “any subsequent unplanned inpatient admission to any acute care facility which occurred within 30, 60, and 90 days of discharge following the qualifying total joint operations.”

^b^Revisit rates were defined as “any visit to an acute care facility which occurred within 30, 60, and 90 days of discharge following qualifying total joint operations bedsides unplanned inpatient admissions—namely, emergency department visits, unplanned, unscheduled outpatient visits, and observation status visits” [11].

^c^The italicized values are statistically significant at a level of .05.

#### Length of Stay

The average length of stay for inpatient hospitalization for the nonparticipating group was 1.87 days as compared with 1.50 days for the participating group (*P*<.001).

#### Patient Engagement

There were 39 unidirectional text messages over 50 days. Patients read their text messages 90% of the time (median), indicated by a read receipt. Of the 39 messages, patients tended to be most inclined to read text messages that included tips on hip care precautions, information on when to stop medications, and what to do the night before surgery. The text message patients were least inclined to read included information on getting presurgery clearance, smoking cessation, and preadmission testing (Table 1).

There were 29 unidirectional emails over the 50-day period. Patients read emails 89% of the time. The email messages patients read the most were getting to know your health care team, planning for your return home, commonly asked questions and answers, and pain medication details. The emails that patients read the least involved information about what surgery entails (videos) and infection and blood clot prevention strategies.

There were 13 bidirectional text messages over the 50-day pathway. Patients responded to messages 54% (median) of the time. Patients were most inclined to respond to questions about their mobilization and incision sites. They were least likely to respond to questions about whether they obtained presurgical clearance, registered for preoperative educational classes, or if they scheduled an appointment with preadmission testing.

MAs and nurses were alerted whenever a patient responded to a bidirectional question in a concerning way, typically because a patient responded to a question on pain levels in a way that indicated severe pain or, alternatively, the patient indicated unusual redness or excessive bleeding at the incision site. The inbound messages to MAs and nurses were minimal, suggesting that patients rarely responded to a bidirectional question in a concerning way. Each office received an average of 2 notifications each week for all of their patients.

#### Patient Experience

With regard to the medication questions on the HCAHPS survey, there were 428 responses to medication questions in the nonparticipating group, with an average of 56% of patients reporting that hospital staff *always* discussed side effects in a way that patients could understand, and an average of 86% of patients reported that the hospital staff *always* described the purpose of new medications (*P* values can be found in Table 5).

**Table 5:** Patient experience analysis: Hospital Consumer Assessment of Healthcare Providers and Systems.

Variables (average %)	Participating (average %)	Nonparticipating (average %)	*P* value	Estimate average difference (nonparticipating-participating)	95% CI
Staff described the medicine’s side effects, mean (SD)	57.95 (49.42)	55.95 (49.70)	.55	−2.00	−4.50 to 8.50
Staff told the patient what the medicine was for, mean (SD)	84.41 (36.32)	85.71 (35.03)	.58	1.30	−5.95 to 3.34
Received information (symptoms to look for), mean (SD)	98.55 (11.98)	96.64 (18.04)	.02	−1.91	0.26 to 3.55
Talked about help needed at home, mean (SD)	96.80 (17.61)	95.17 (21.46)	.13	−1.63	−0.47 to 3.74

There were 485 responses to the same question from the participating group, with an average of 58% of patients reporting that the hospital staff *always* discussed medication side effects in a way patients could understand, and an average of 84% of patients reported that hospital staff *always* described the purpose of new medications.

The composite score for the medication questions was 71.3% for the participating cohort and 71% for the nonparticipating cohort.

#### Patient Satisfaction With the Mobile Health Technology

A total of 75/2059 (0.4%) patients *opted-out* of the mHealth technology program after enrollment by texting *stop* in response to text messages, all of whom opted-out in the postdischarge window, and usually did so toward the end of *the pathway*. We asked patients using mHealth technology whether they found it helpful to receive text messages, reminders, and emails by way of the mHealth technology from their health care providers. A total of 580 of the 2059 patients (28%) responded to the satisfaction question; 390 (67%) responded that they strongly agree that the participation was helpful; 166 patients (29%) responded that they agree that the mHealth technology was helpful; 10 patients (1.7%) were undecided; and 8 patients (1.4%) disagreed that mHealth technology was helpful. Only 6 patients (1%) strongly disagreed.

## Discussion

### Principal Findings

We found that patient-facing mHealth technology may positively impact patient readmissions and length of stay in significant ways, even after a few months of implementation of the technology. Patients cared for by participating surgeons were readmitted at a significantly lower rate as compared with patients of nonparticipating surgeons within 30, 60, and 90 days after surgery in both the subset analysis (Multimedia Appendix 1) and in the full analysis presented in the Results section (January 1-December 31, 2019). Furthermore, the length of hospital stay for patients cared for by the participating surgeons was about one-third less than that for patients of nonparticipating surgeons in both the subset and full analyses. We also found significant improvements in patient-centered care measures in the subset and full analyses.

The importance and significance of these findings are remarkable given that mHealth technologies are considered a way of the future, and have been shown to be remarkably popular and essential during the COVID-19 pandemic [4,22-24]. Our findings run counter to the notion that after technology adoption, the reduction of readmission rate will necessarily take months or years to achieve or that they cannot be sustained over time [25,26].

Of note, we found differences in the comorbidities, suggesting that patients cared for by participating surgeons were *sicker* than patients cared for by nonparticipating surgeons, at least at the time of enrollment in the mHealth technology. This finding is interesting in that if there were patient selection biases present, one might expect that health care professionals would favor enrolling *healthier* patients on mHealth technology [16,27-29]. Unfortunately, the opt-out rate for patients cared for by participating surgeons was too small of a cohort to discern whether there were any differences between the opt-in versus opt-out patient cohorts in the participating surgeon group. It is possible that the sicker patients of the participating surgeons were self-selecting to participate, which can be validated by future research.

We also found that mHealth technologies can impact multiple outcomes at the same time, including clinical outcomes and patient-centered care measures. These findings lend support to the notion that patient-centered care processes likely enhance several quality dimensions simultaneously [2]. Specifically, although existing empirical evidence suggests there may only be a modest positive association between patient-centered care and clinical outcomes [11,12,18,19,30], there is still a strong reason to use a systems-based approach to improve multiple aspects of care quality. Quality improvement efforts aimed at enhancing patient-centered care might improve infrastructure and processes, resulting in broader quality of care improvements on multiple levels [20].

The broad experience of successful use of mHealth technologies during the COVID-19 pandemic shows that effective technologies go beyond merely monitoring purposes by integrating the informational and educational needs of the patient/their family/caregivers. Indeed, we theorize that the primary reason for the shorter length of stay for patients in the participating cohort is by virtue of the educational benefits of mHealth technology, that is, the patients might have better understood the importance of early mobilization and/or how to take care of themselves at home. This is a prime example of coproduction in which patients actively change their behavior to achieve better outcomes [31]. The patients benefiting from the mHealth technology might have been more *activated* because of the digital preparation and education materials when compared with patients in the nonparticipating cohort. Our theories about what patients are thinking, however, are speculative and will need to be validated by future research.

Another significant contribution of this research is demonstrating how we were able to quantify patient engagement by how often they read and responded to messages and assessing the types of messages that patients preferred [29]. There was a distinct pattern associated with the types of messages and content that patients chose to be engaged with. In this study, patients were least responsive to prompts where the hospital was asking them to *do* something (active); they were most engaged with emails that were fact-based informational emails (passive). Perhaps patients perceive adherence questions as overly intrusive. Future research needs to be done using mHealth technologies in a co-designed process to better discern patients’ thought processes on adherence [31].

This study has several limitations. The *participating* cohort consisted only of people who agreed to participate because (1) we could not ethically randomly withhold technology from patients who, based on the feedback they provided us during the co-design phase, overwhelmingly wanted to have access to and be monitored on the mHealth technology and (2) we could not ethically require nonagreeing patients to engage in substantive, recurring actions on a repeated basis—namely, reading and responding to messages every day for 50 days. Therefore, randomization was not feasible from an equipoise standpoint [32].

However, by including only patients who agreed to use mHealth technology, we acknowledge a significant methodological flaw, a selection or Berksonian bias that is often inherent in studying digital interventions—a phenomenon that arises when the sample is taken not from the general population but from a preselected subpopulation [33]. Patients who agree to participate are generally more motivated, have greater self-efficacy, are more literate, and have a variety of other attributes that make it likely that their outcomes would be better than nonagreeing patients [34-37].

To offset these limitations, we used a combination of different analytic methods, not only to address the issue of patient selection bias per se but rather to show the durability of our results and, by extension, their external generalizability to the larger population. We arrived at the same conclusions through different analytic approaches and ensured that the conclusions drawn are consistent. Specifically, we found consistent outcomes at different time intervals separated by several months. We also found that less than 1% of patients declined to participate or opted-out of participating after enrollment, and few patients indicated a preference for a language other than English in both the participating (43 patients) and nonparticipating (119 patients) surgeon groups [37].

It could also be argued that, by excluding people who preferred a language other than English, we could have unintentionally exacerbated health disparities. After all, recent research focusing on mHealth technologies has linked their implementation to enhanced risk for racial bias and health disparities, rather than adhering to the promise of equalizing health inequities [38]. However, it is important to recognize that there were too few patients in this study with a preference for a language other than English such that it would be impossible to discern differences between the populations in the nonparticipating and participating groups based on language. It would be most methodologically sound to exclude patients with a preference for a language other than English to ensure that the populations in both cohorts were similar to reduce the risk of introducing a confounding variable. Limiting this feasibility/pilot study to patients with an English preference helped control for *translation* variables. Furthermore, as demonstrated in Table 2, ethnicity and race were nearly identical for patients cared for by participating and nonparticipating surgeons alike.

We acknowledge that one cannot draw mechanistic conclusions from an observational study. Thus, we applied and interpreted the Bradford Hill criteria to support our causal inference in evaluating the data [39,40]. Bradford Hill recognized that most epidemiological research, like in our study, is conducted in nonexperiential, inherently real-world environments in free-living populations [39,41]. Bradford Hill proposed several different aspects of associations for evaluating traditional epidemiological data, including the strength of the association, consistency, biologic gradient, and temporality. They argued that a single study, no matter how statistically sound, cannot prove causation [39]. However, consistency in association throughout a variety of different methods should be viewed as compelling and likely indicative of a causal connection [41].

Stated differently, our results cannot speak to the efficacy of mHealth technologies because of patient selection biases and other design limitations inherent in observational studies. However, by using a combination of different analytic and robust methods, all of which show similar outcomes over time (ie, meeting Bradford Hill’s temporality, consistency, biologic gradient, and strength of the association criteria), we can say with a high degree of confidence that the durability of our results, coupled with the observation that a high proportion of patients agreed to participate speaks to the robust external generalizability and effectiveness of our findings.

Finally, some of our findings relate to patients’ self-reported measures, which are known to have their own limitations. For instance, in language preference and patient satisfaction in our study, patients report (and we rely on) their own stated preferences, which may not represent their true feelings [42]. Patients could be susceptible to well-documented social desirability biases, where patients are inclined to choose a higher rating for patient satisfaction or English as their preferred language to appear more favorable or likable to themselves or others [42]. We also cannot state any definitive claims about patient satisfaction because of the low response rate to the embedded question on whether and how much patients liked the mHealth technology.

### Conclusions and Future Directions

We demonstrated that patient-facing technologies that empower patients and their caregivers to become involved and informed in their care and, specifically, to play a more active role in enhancing patient care, can be effective. This is supported by the growing movement to embrace the potential of mHealth technology to transform health care outcomes, a movement of which is becoming increasingly pronounced and urgent with COVID-19 developments [7]. The purpose of this observational study was to assess whether a multicomponent mHealth technology impacts clinical outcomes and/or enhances patient-reported outcomes. We provided actionable data to demonstrate that mHealth technologies can be effective, which can help support and shape how patient-facing mHealth technology is being used during and following COVID-19.

This study should serve as a foundation for future research. Recent research has shown that technology-based implementations can be susceptible to the *digital divide*, which coexist with other social determinants of disparity by inadvertently masking or exacerbating racial, ethnic, or gender inequities [43]. Thus, future research needs to assess the hypothesis that mHealth technologies will improve, and certainly not widen, existing disparities by systematically examining nonrandom biases (such as health access issues) and how that might impact data.

Furthermore, there have been no studies published, to our knowledge, regarding the comparative effectiveness or efficacy of mHealth technologies [2,24]. It is currently unknown whether certain mHealth technologies, including some already in use, are effective or are more impactful than others. Further studies are also warranted to examine the impact mHealth technologies have on clinical workflows and resource utilization [24]. We demonstrated that our results were sustainable for several months, but longer research related to sustainability beyond 1 year is needed. Greater systematic evaluation and research, including prospective, multisite studies (if possible), are needed to fully characterize the effectiveness of digitally facing patient-centered technologies.

## Appendix

Multimedia Appendix 1 Results from January 1, 2019 to April 30, 2019.

## Abbreviations

COVID-19: coronavirus disease 2019
GEE: generalized estimated equation
HCAHPS: Hospital Consumer Assessment of Healthcare Providers and Systems
MA: medical assistant
mHealth: mobile health
OR: odds ratio
TJR: total joint replacement
