# Supplementary material for: Assessing the Impact of Patient-Facing Mobile Health Technology on Patient Outcomes: Retrospective Observational Cohort Study
Source: JMIR Mhealth Uhealth. 2020 Jun 26;8(6):e19333. doi: 10.2196/19333 (PMC7381069; doi:10.2196/19333)
Supplement: Multimedia Appendix 1 [file mhealth_v8i6e19333_app1.docx]

| **Variables** | **NPS** | **PS** | ***p* Value** |
| --- | --- | --- | --- |
| Mean ± standard deviation age (year) | 68.65 ± 9.50 | 68.05 ± 10.08 | 0.161 |
| Sex |  |  |  |
| Female | 58.49% | 58.17% | 0.984 |
| Male | 41.51% | 41.83% | 0.984 |
| Race |  |  |  |
| White | 82.68% | 84.06% | 0.673 |
| African American | 11.59% | 9.96% | 0.543 |
| Asian | 2.36% | 0.80% | 0.192 |
| Native American | 0.22% | 0.00% | 1 |
| Others | 1.01% | 2.39% | 0.168 |
| Ethnicity |  |  |  |
| Hispanics | 8.10% | 7.57% | 0.887 |
| Non-Hispanics | 90.44% | 92.03% | 0.517 |
| Declined | 1.46% | 0.40% | 0.304 |
| Comorbidities |  |  |  |
| Hypertension | 38.00% | 35.00% | 0.965 |
| Hyperlipidemia | 31.00% | 25.00% | **0.001** |
| Gastro-esophagus reflux disease | 34.00% | 30.00% | 0.442 |
| Mean ± standard deviation length of stay (days) | 1.94 ± 2.14 | 1.43 ± 1.20 | **<0.001** |

***SUPPLEMENTARY FILE:***

This file contains an analysis of the first few months the digital technology was implemented (January 1, 2019-April 30, 2019). The Results, as you can see below, are similar the full analyses represented in the Results of the manuscript (January 1, 2019-December 31, 2019).

**Table 1.** Sample Characteristics of Patients Cared for By Non-Participating (NPS) Surgeons and Patients Cared for by Participating (PS) Surgeons for January 1, 2019-April 30, 2019 (N= 889 Non-Participating Patients; n= 251 Participating Patients). P values in bold are statistically significant.

**Table 2:** Summary Characteristics of Non-Participating (NPS) Surgeons and Participating (PS) Surgeons for January 1, 2019-April 30, 2019 (n=35 Non-Participating Surgeons; 12 Participating Surgeons)

| Surgeon Results |  |  |  |
| --- | --- | --- | --- |
| **Variables** | **PS** | **NPS** | ***P-Value**** |
| Age (years) | 55.5±12.29 | 56.83±17.64 | 0.872 |
| Experience (years) [Median, IQR] | 22.5 [11.00,28.00] | 26.00 [10.25,32.75] | 0.630 |
| Total joint replacement annual case volume[Median, IQR] | 187.5 [138.00,222.00] | 114.50 [66.00,157.00] | 0.149 |
| Readmission rates [Median, IQR] | 2.85 [1.87, 4.03] | 2.55 [0.67, 3.64] | 0.631 |
| Patient satisfaction scores [Median, IQR] | 91.50 [81.75,97.50] | 91.50 [87.50, 98.50] | 0.808 |

***P value was calculated at the 0.05 level of significance.**

**Table 3.** Readmissions* and Revisit Rates** Analysis of Patients of Non-Participating (NPS) Surgeons and Patients of Participating (PS) Surgeons for January 2019-May 2019

| Outcome Variable | NPS | PS | *P* Value |
| --- | --- | --- | --- |
| **Hospital readmission** within 30 days | 4.7% | 1.6% | **0.026** |
| 60 days | 7.4% | 3.2% | **0.016** |
| 90 days | 10% | 3.6% | **0.001** |
| **Emergency department** visits within 30 days | 4.3% | 3.6% | 0.698 |
| 60 days | 6.4% | 4.7% | 0.419 |
| 90 days | 7.2% | 4.8% | 0.226 |
| Unplanned, unscheduled **outpatient visits** within 30 days | 0.8% | 1.9% | 0.193 |
| 60 days | 2.8% | 4.0% | 0.460 |
| 90 days | 4.8% | 8.7% | **0.044** |
| **Observation status** visits within 30 days | 1.9% | 1.2% | 0.622 |
| 60 days | 2.7% | 2.0% | 0.687 |
| 90 days | 3.1% | 2.8% | 0.931 |

* Hospital readmissions were defined as any subsequent unplanned inpatient admission to any acute care facility which occurred within 30, 60, and 90, days of discharge following qualifying total joint operations.

** Revisit rates were defined as any visit to an acute care facility which occurred within 30, 60, and 90, days of discharge following qualifying total joint operations bedsides unplanned inpatient admissions—namely, emergency department visits, unplanned, unscheduled outpatient visits, and observation status visits.^11^

P values in bold are statistically significant.

**Table 4:** Patient Experience Analysis: Hospital Consumer Assessment of Healthcare Providers and Systems (HCAHP)

*Patients of Non-Participating (NPS) Surgeons and Patients of Participating (PS) Surgeons for January 2019-May 2019*

| **Variables (Average %)** | **PS (Average %)** | **NPS (Average %)** | ***p-value*** |  |
| --- | --- | --- | --- | --- |
| Staff Described Medication Side-effect | 61.76 | 48.44 | 0.212 |  |
| Told Patient What Medicine Was For | 88.24 | 81.25 | 0.378 |  |
| Received Information (Symptoms to look for) | 95.65 | 95.96 | 0.932 |  |
| Talked about help needed at home | 93.48 | 92.00 | 0.757 |  |

| **Variables (Average %)** | **PS**  **(Average %)** | **NPS (Average %)** | ***P-value*** | **Estimate Average Difference {Non-participating-Participating}** | **95 % CI** |
| --- | --- | --- | --- | --- | --- |
| Staff Described Med Side-effect | 61.76 | 48.44 | 0.212 | -13.33 | [-34.13, 7.47] |
| Told Patient What Medicine Was For | 88.24 | 81.25 | 0.378 | -6.99 | [-22.45, 8.49] |
| Received Information (Symptoms to look for) | 95.65 | 95.96 | 0.932 | 0.31 | [-6.70, 7.32] |
| Talked about help needed at home | 93.48 | 92.00 | 0.757 | -1.48 | [-10.76,7.80] |
